# Retention Assessment of High Performance Poly-etheretherketone Removable Partial Denture Frameworks Constructed by Various Techniques (*in vitro* Study)

**DOI:** 10.30476/DENTJODS.2021.87488.1265

**Published:** 2021-12

**Authors:** Nesreen El Mekawy, Mohamed Elgamal

**Affiliations:** 1 Dept. of Prosthodontics, Faculty of Dentistry, Mansoura University, Mansoura, Egypt

**Keywords:** Polyetheretherketone, Dental Prosthesis Retention, Removable Partial denture

## Abstract

**Statement of the Problem:**

Poly-etheretherketone is a novel material used in the construction of the removable partial dentures frameworks instead of the metal frameworks. This material can be fabricated by various techniques. Most common methods are the injection molding or Computer Aided Design/Computer Aiding Manufacturing (CAD/CAM) milling techniques. The fabrication technique may affect the adaptation of the frameworks by influencing the retention.

**Purpose:**

To assess the effect of the processing techniques of high performance Poly-etheretherketone either by injection molding (pressing) or CAD/CAM milling techniques on removable partial denture frameworks retention for rehabilitation of upper class I Kennedy classification.

**Materials and Method:**

This *in vitro* study was performed on one epoxy resin model representing the partially edentulous maxillary arch with natural teeth extending from first premolar to first premolar. First premolars and canines were reduced to receive porcelain fused to metal crowns with 0.50mm mesio-buccal retentive undercuts, distal guiding planes and mesial occlusal rest seat on first premolars and cingulum rest seat on canines. Considering the construction technique of frameworks, twenty samples were divided into two groups. In the group I, ten frameworks were fabricated by injection molding, and in the group II, ten frameworks were fabricated by CAD/CAM. The removal and insertion was carried out at 120, 720 and 1440 cycles for both groups, respectively. The retention values were measured by using Universal Testing Machine before cycling and after each interval.

**Results:**

Independent t-test showed significant difference on retention at different simulation cycles between groups. Group II exhibited significantly less retention than group I (*p*< 0.001), while comparing the retention at different cycles within each group by paired sample t-test exhibited significant decrease of retention till the end of the cycling (*p*< 0.001).

**Conclusion:**

From the retention point of view, high performance poly-etheretherketone frameworks fabricated by injection molding technique provided a promising method over CAD/CAM technique milling method.

## Introduction

Nowadays, due to continuous development and advancement in polymer’s industry fabrication, numerous materials have been introduced for dentures construction [ 1 ]. Currently, high performance thermoplastic polymer polyetheretherketone (PEEK) has obtained increasing attention for dental applications; PEEK shows low density, good biocompatibility and provides dampening properties as well as certain ductility [ 2 ]. Moreover, it is characterized by high heat resistance, high mechanical strength, and has a maximum degree of crystallinity (48%) and the low solubility (0.5%) and water absorption values under different aging solution [ 3 ]. 

High performance polyether-etherketone (PEEK) polymer (BioHPP) is used in construction of maxillary class I Kennedy classification removable partial dentures (RPDs) frameworks; it helps in diminishing the stresses and distal torque on abutment teeth [ 4 ]. Additionally, it achieves patient’s desire of metal free RPDs and avoids the risk of oral galvanism [ 5 ]. BioHPP is a practicable biomaterial, which allows the replacement of conventional polymers, as well as the replacement of metals, ceramics, and alloys in the dentistry field.

BioHPP ^®^ materials are available in both granulate and pellet form for injection molding technique also they are obtainable as milling blanks for the Computer Aided Design/Computer Aiding Manufacturing (CAD/CAM) technology. Nevertheless, in the field of dentistry, BioHPP may be processed by utilizing the well-known thermoforming technology [ 6 ].

In the last decade, great accomplishments were obtained particularly in prosthetic branch of dentistry concerning CAD/ CAM technology [ 7 ]. CAD/CAM technology with different techniques of production leads to the improvement of RPDs quality, furthermore, it increases the manufacturing efficiency outcomes [ 8 ]. Generally, BioHPP shows good milling properties and it is commonly the most used material in CAD/CAM technology [ 9 ]. This technology has no processes of wax pattern, investment, and casting, thereby avoiding oxidation firing cycle and allowing normal cooling rate. It enables the holistic molding of RPDs frameworks via optimization of undercut position and size [ 10 ].

Injection molding (pressing) technique is one of the commonly utilized fabrication technologies in plastics industry [ 11 ]. In semi-crystalline polymer materials such as BioHPP, the temperature of mold is a corner stone in defining the parameters of the injected product for performance; BioHPP revealed high strength than numerous metals on a per mass basis. Previous study has suggested utilizing an exemplary temperature of the metal mold 175–220° C for semi-crystalline PEEK polymer [ 12 ]. Nevertheless, in the dental application, utilization of refractory products to form the mold is commonly used method to construct a positive reproduction for arches and associated structures [ 13 ].

Sufficient retention of RPDs is considered as one of the most important factors affecting their clinical success. Retention of RPDs is accomplished by placing clasp parts into undercuts on abutment teeth [ 14 ]. Some patients are not satisfied with their RPD, especially when it is not stable during mastication [ 15 ]. A previous study [ 16 ] investigated the initial retentive force of a BioHPP clasps to an abutment tooth; it was reported that retention force was less than that of chrome cobalt RDP frameworks. This is a matter of concern, especially with the use of a shallow under cuts equal to 0.25 mm ,which is the most routinely used depth of undercut, hence, it is essential to investigate the initial retentive force and retentive force using BioHPP clasps. 

BioHPP materials have the prospect to engage and disengage undercuts without being stressed beyond their elastic limit. This opens up the possibility of prolonged and improved retention (over many years) for the RPDs frameworks fabricated from these novel materials. The *in vitro* repeated removal and insertion of frameworks, represents the simulation of many years of use. It also allows for a comparison of frameworks deformation following prolonged removal and insertion testing, which would be expected to have an undesirable effect on frameworks retention [ 17 ].

The aim of the current *in vitro* study was to assess and compare the effect of BioHPP processing techniques by either injection molding (pressing) or CAD/CAM milling technique on retention of RPDs frameworks used to rehabilitate maxillary class I Kennedy classification with a specific end goal to examine their retentive force. The null hypothesis was that the processing techniques have insignificant influence on the retention of BioHPP RPDs frameworks.

## Material and Method

This *in vitro* study was performed on an epoxy resin study model to improve the standardization of the study, which represents maxillary class I Kennedy classification with teeth elongating from the first premolar on one side to the first premolar on opposite side. On this epoxy resin study model, twenty BioHPP maxillary Kennedy class I RPDs frameworks were constructed; ten frameworks by CAD/CAM milling technique, and ten frameworks by injection molding technique. Retention values were evaluated for each BioHPP framework. 

### Fabrication of the Study Model

Epoxy resin study model was constructed through the duplication of commercially available maxillary Kennedy class I model (BH303; Nissin Dental Products Inc., Kyoto, Japan). A silicon rubber base impression material (Speedex Coltene/Whale dent .Inc. Cuyahoga Falls, Ohio 44223/ USA)was used to produce a mold on which the epoxy resin (Bredent GmbH & CO. KG weissenhorner Straße. Senden, Germany) was poured to fabricate the model. 

**Figure 1:**
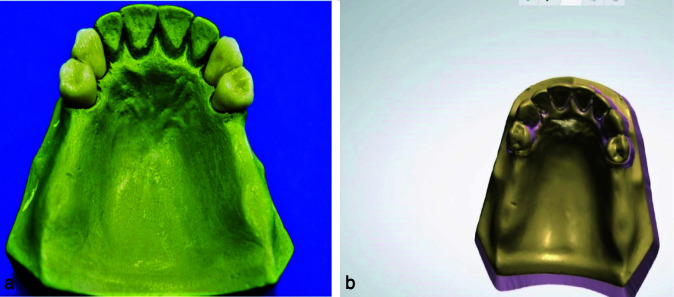
**a:** Epoxy resin study model with porcelain crowns cemented over prepared abutment teeth. **b:** 3D virtual model exported as STL file format

Both first premolars and canines teeth on each side of the epoxy resin model were reduced to obtain full porcelain fused to metal (PFM) crowns on each tooth. These crowns were fabricated with 0.50mm mesio-buccal retentive undercuts, distal guiding planes and, mesial occlusal rest seat on first premolars abutment teeth, and cingulum rest seat on canines (Figure 1a). The fabrication and cementation of the PFM crowns on the prepared teeth of the epoxy resin model were done.

### Scanning the Master Model

Epoxy resin study model (master model) was fixed on the table of 3 Shape scanning machine dental system, after application of scanable spray (Telescan Spray zur vorbereitung von oberflächen) over the stone teeth and PFM crowns of master model. The blue LED and multi-line scanning was used to capture 140000 points on the surface of the master model to form point cloud. A 3D virtual model was created by connecting these points cloud together into triangular facets; lastly, the 3D virtual model was exported as STL file format (Figure 1b) to be ready for wax design of RPDs frameworks using CAD technology.

### CAD Design of RPDs Frameworks

**Figure 2:**
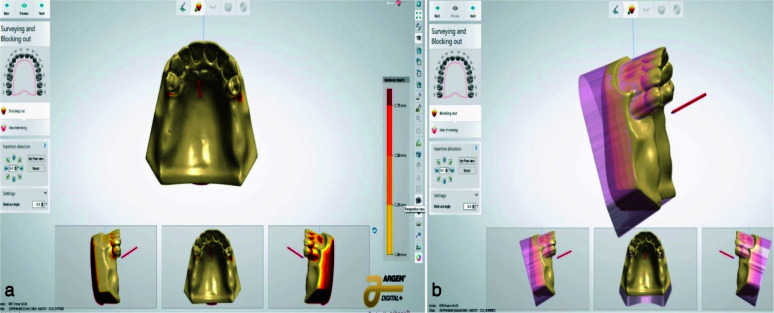
**a:** Surveying of 3D virtual model by digital surveyor to determine the path of insertion and removal, undesirable undercut marked by red color, **b:** Blocking out the undesirable undercut

3D virtual model was imported into the CAD software (3shape dental system; 3Shape A/S, Copenhagen, Denmark) to start the designing process. It was surveyed by digital surveyor to determine the path of insertion and removal (Figure 2a). 3D virtual model undercuts were identified with different colors on the model surface (dark red color means more than 1.5mm, red 1 to 1.5mm, orange 0.5 to lmm, yellow 0.25 to 0.5mm and light yellow 0 to 0.25mm). The undercuts marked by red color were undesirable undercut thus; it was blocked out (Figure 2b). The design of RPDs framework was started by the CAD software (3shape dental system; 3Shape A/S, Copenhagen, Denmark).

**Figure 3:**
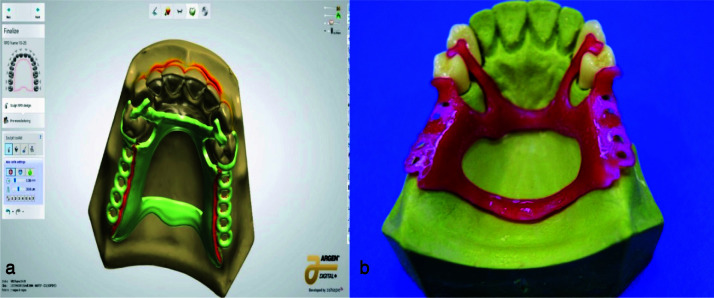
**a:** Final CAD design of High performance PEEK framework. **b:** Tentative resin frameworks constructed by rapid prototyping technique

For all samples of the study, a symmetrical design of the maxillary RPD frameworks was considered. The design included antero-posterior palatal bar major connectors, connecting 2- distal extension denture bases with external finish line. The proximal plate extended from the denture bases on the distal surface of abutment teeth (first premolar) with two thirds from marginal ridge to cervical line length to carry modified Aker clasp arm on buccal surface of premolars teeth that engage mesio-buccal retentive undercuts of 0.50mm depth, created on the PFM crown cover the premolar tooth. The minor connectors were placed between canine and first premolar abutment teeth to carry the cingulum and occlusal rests (Figure 3a). The framework components were located in the correct position in the form of connected dots. 

The width and thickness of all parts were converting into a solid volume. The surfaces were thickened by shell tool to form 2mm thickness in outside direction. All the angles were smoothed and borders were carved to adjust the antero-posterior palatal bars. The whole framework design was virtually checked from all surfaces. The tentative resin frameworks were constructed by utilizing rapid prototyping technique to examine the accuracy of the frameworks adaptation to the epoxy master model of the study (Figure 3b).

In this study, there was no need to use conventional chrome cobalt RPDs as a control group as previous studies [ 16 , 18 ] established that BioHPP exerts fewer stresses on abutments compared to standard-alloy clasps, provides adequate retention, and satisfies aesthetic demands. These issues indicate that BioHPP presents a promising alternative to conventional metal clasps. Therefore, according to the technique utilized in the construction of BioHPP RPDs frameworks, the twenty frameworks were divided into two equal groups. In the group I (n=10), BioHPP RPDs frameworks were fabricated by injection molding technique, and in the group II (n=10), BioHPP RPDs frameworks were fabricated by CAD/CAM technique.

### Construction of BioHPP RPDs Frameworks by Injection Molding Technique

**Figure 4:**
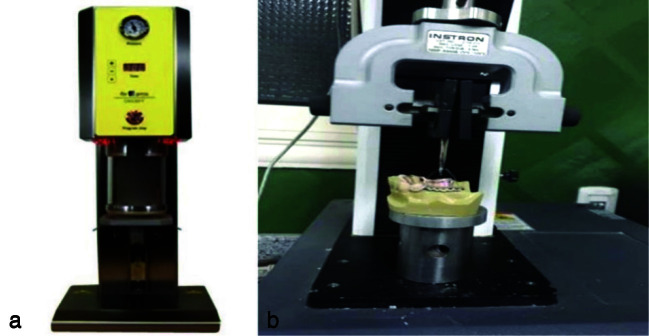
**a:** For 2 Press System, **b:** Pulling chains to permit the pullout of Removable partial denture frameworks for retention measuring

Ten BioHPP RPDs frameworks were constructed by injection molding technique [ 4 , 19 ] and for 2 Press System (Figure 4a) in the following procedures.

The fitting of the ten 3D printed resin frameworks on the epoxy model were assessed and then the master model was duplicated into ten refractory cast to be used during the construction of the RPDs frameworks. Each printed resin frameworks was adapted to a refractory cast and the sprues were connected to it to act as a path of the melting BioHPP. Then, it was invested in a special silicon ring with a special phosphate-bound investment material (Brevest investment material For 2 Press System). The mold was pre-heated up to 630°C-850°C, for removing of the trial framework and then cooled at 400°C to reach the melting range of BioHPP material. Following the complete melting of BioHPP granules, the press plunger was imported in the reservoir of the cylinder. The procedure was fully completed within 35 minutes; the mold was left to cool and divested or uncovered as usual. The BioHPP frameworks were disconnected from the sprues, and then they were finished and polished in regular manner.

### Construction of BioHPP RPDs Frameworks by CAD/CAM Technique

The other ten BioHPP RPDs frameworks were constructed by CAD/CAM technique in the following steps [ 16 , 20 ]. Following the assessment of the 3D printed resin frameworks on the epoxy resin model, for each framework of the ten samples, the 3D design was introduced directly to manufacturing compartment to begin the milling process of BioHPP dental discs (BioHPP, Bredent GmbH) by using Ceramill motion 2 milling machine (Ceramill Motion 2, Amann Girrbach). The BioHPP framework was then removed from the disc by using carbide bur, then smoothed by removing any sharp angles and finally finished and polished in usual manner.

### Evaluation of Retention Values of Both Groups Frameworks

The retention of the BioHPP RPDs frameworks was measured initially (0 cycles), then the retention was measured after being subjected to 120, 720 and 1440 insertion/ removal test cycles (ROBOTA chewing simulator integrated with thermocyclic protocol operated on servo-motor (Model ACH-09075DC-T, AD-technology CO., LTD., Germany) [ 21 ]. These cycles were corresponding to one month, six months and, one year, respectively by Universal Testing Machine (Shimazdu testing machine AG-X, 10N-10KN, Japan). This measurement was done by applying dislodging forces to the center of the frameworks in a vertical direction.

Three holes were made in anterior and posterior bar of major connector to allow attaching three pulling chains that were secured to metal ring hook to permit the pullout of BioHPP RPDs frameworks (Figure 4b). This enabled the placement (insertion) of the framework to its predetermined terminal position and its subsequent removal from this position by Instron Testing Machine. The maximum loads required to remove the RPDs frameworks at 0, 120, 720 and 1440 continuous cycles (corresponding to 0 as a base line, 1 month, 6 months and 12 month of simulated clinical use of a RPDs denture) were recorded by computer software. The mean values of the retentive force magnitudes were recorded 5 times for dislodgement of each framework at each corresponding cycle [ 21 ]. 

### Statistical Analysis of the Recorded Data

Data were tabulated, coded, and then analyzed using the computer program SPSS (Statistical package for social science) (SPSS Inc. Chicago IL, USA) version 23.0.

The descriptive statistics were calculated in the form of Mean± Standard deviation (SD). In the statistical comparison between both groups, the significance of difference was examined by using Student's t-test to compare the mean values of both groups. A *p* Value<0.05 was considered statistically significant.

## Results

Table 1 reveals the descriptive statistics of mean retention values for the BioHPP RPDs frameworks constructed by either injection mold technique or CAD/CAM milling technique at 0, 120, 720 and 1440 insertion/removal continuous cycles (which correspond to: 0 as a base line, 1 month, 6 months and 12 month of simulated clinical use of RPDs). It was found that the highest retention values were at 0 insertion/removal cycle, whereas; the lowest retention values were corresponding to 1440 insertion/removal cycle for both groups of the study. In addition; a comparison of mean values between each cycle retention values within each group using paired t- test (Table 1) showed that the retention value for the group I and group II was significantly decreased by time (*p*< 0.001 ).

**Table 1:** Comparison between retention values of PEEK framework at different cycles within each group

		At o cycle	At 120 cycles	At 720 cycles	At 1440 cycles	*P*
Group I	X`	5.38	5.17	4.66	4.26	< 0.001*
	±SD	±0.20	±0.14	±0.15	±0.40	
Group II	X`	3.40	3.20	2.85	2.55	< 0.001*
	±SD	±0.21	±0.18	±0.15	±0.14	

X`:Mean, SD: standard deviation P:Probability

* :significance <0.05 Test used: Paired t- test

Table 2 shows the Student t-test for comparing the retention values of BioHPP RPDs frameworks constructed with either injection mold technique or CAD/CAM milling technique. It was found that at 0 insertion/ removal cycle, the BioHPP RPDs frameworks constructed by direct injection molding (pressing) technique showed statistical significant high retention value (5.38±0.20 N) than the BioHPP RPDs frameworks constructed by CAD/ CAM milling technique (3.40±0.21 N), as *p*= 0.000687. However, at 120 insertion/removal cycles, group I (BioHPP RPDs frameworks constructed by direct injection molding (pressing) technique) showed a significant high retention value (5.17±0.14 N) than group II (BioHPP RPDs frameworks constructed by CAD/CAM milling technique) (3.20±0.18 N), as *p*= 0.000303. In addition, at 720 insertion/removal cycles, group I recorded a statistically significant higher retention mean value (4.66±0.15N) than group II (2.85±0.15 N), as *p*= 0.000243. Moreover, at 1440 insertion/ removal cycles, it was found that group I presented a significant higher retention mean value (4.26±0.40N) than that of group II (2.55±0.14 N), as *p*= 0.004. On both groups, there was a statistically significant difference between the retentive forces of the BioHPP RPDs frameworks during all insertion/removal cycles. BioHPP RPDs frameworks exhibited the highest retention at 0 cycle. In addition, there was significant retention decrease at all insertion/ removal cycles 120, 720 and 1440 cycles, that corresponds to 1 month, 6 months and one year respectively.

**Table 2:** comparison of the retention values of High performance PEEK RPDs frameworks constructed with either injection mold technique or CAD/CAM milling technique

	Variable	Mean X`	± SD	Statistics *p* Value
At 0 cycle	Group I	5.38	±0.20	*p*= 0.000687^**^
	Group II	3.40	±0.21	
At 120 cycle	Group I	5.17	±0.14	*p*= 0.000303^**^
	Group II	3.20	±0.18	
At 720 cycle	Group I	4.66	±0.15	*p*= 0.000243^**^
	Group II	2.85	±0.15	
At 1440 cycle	Group I	4.26	±0.40	*p*= 0.004^*^

## Discussion

This *in vitro* study was designed to evaluate and compare the retention values of BioHPP RPDs frameworks constructed by injection mold technique with CAD/ CAM milling technique after insertion/ removal simulation cycle at the end 1440 cycles (equivalent to one year of patient use). The results of the study rejected the null hypothesis that the processing technique has in significant influence on the retention of BioHPP RPDs frame- works. Torqueing of the clasped teeth and possible traumatization of residual alveolar ridge is a common sequel of tissue-ward movement of the tooth–mucosa–borne prosthesis [ 22 ]. Frameworks fabricated from PEEK are advantageous for the health of periodontal tissues of the abutment teeth since the PEEK elasticity might reduce the stress on the abutment teeth and the distal torque [ 23 - 24 ]. BioHPP has been proven a successful alternative for the fabrication of RPD frameworks. PEEK has a modulus of elasticity resembled to the bone tissue with 4 GPa. Consequently, this will reduce the stress on the abutment teeth. Furthermore, the PEEK material color provides appearance that is more aesthetic. PEEK clasps have more gentle effect on the enamel tissue and the PFM restorations when compared to the conventional chrome cobalt clasps. Moreover, the periodontal health of the abutment teeth is maintained due to its low plaque affinity [ 25 ]. It was found that under same loading conditions, PEEK framework demonstrated the best protection function on the PDL, which may be particularly suitable for patients with poor periodontal conditions [ 26 ].

In the present study, both first premolars and canines teeth were prepared to obtain full PFM crowns with mesio-buccal retentive undercuts of 0.50 mm on first premolars teeth. This in accordance with the results of Tannous *et al*. [ 27 ] that reported clasps made from PEEK resulted in a low retentive force than the clasps made from metals. Nevertheless, PEEK clasps that were designed properly and utilized 0.5 mm retentive undercut might produce adequate retention values for clinical use [ 27 ]. In addition, the highly elastic nature and flexibility of PEEK polymer might be beneficial in clasps designing, using deeper retentive undercuts on the remaining teeth, which consequently diminishes the denture pain resulted from excessive local pressure [ 12 ]. The higher undercut has been advocated by other studies [ 27 - 29 ] in order to overcome the lower removal force to dislocate the flexible clasps. 

The abutment teeth (first premolars and canines) were prepared to receive PFM crowns because the porcelain material revealed the least wear amount, followed by enamel. A previous study [ 14 ], improved that the well-constructed glazed porcelain surface is able to withstand the forces of RPDs causing the wear of retentive clasp arms. These results are in agreement with those of Tietge *et al*. [ 30 ] and Marso *et al*. [ 31 ]. 

In this study, the impact of BioHPP RPDs frameworks processing technique on the retention forces was examined. It was found that injection mold frameworks showed higher retention with significant values than CAD/CAM milled frameworks (*p*< 0.05). This may be attributed to the perfectly fitting, adapted prosthesis and controlled friction properties by investment offered by the For 2 Press System used for fabrication of pressed BioHPP RPDs frameworks [ 4 , 19 ]. The burs used for milling in CAD/ CAM technique might had adverse side effect on adaptation of the BioHPP RPDs frameworks, as the milled-PEEK had a low impact strength of 4 kJ/m^2^ [ 12 ]. Thus, this property had an adverse effect on the friction occurred between the PFM crowns and the inner surface of the clasp.

The present study found that in both groups of the study, BioHPP RPDs frameworks clasps exhibited high retention values during the first period of cycling, which could be explained by achieving PEEK clasp requirement for accepted retention by designing clasp with 1.5 mm thickness; moreover, the authors found that deep undercut increased the retention value. This might lead to lowering the ratio between the strength/stress, and the probability of failure decrease [ 32 ]. This current study is in contrary with the study of Bauer *et al*. [ 33 ] who concluded that the method chosen for fabrication of the clasps has a great influence and reported that by fabricating milled clasps with CAD/CAM system, superior values of retention force of up to 8,6 N are reached. 

This *in vitro* study showed that the initial retention value of both group was between 5.38 N for group I and 3.40 N for group II, which is an acceptable retention value for the BioHPP RPDs frameworks clasps. This result is in agreement with Marie *et al*. [ 17 ] who reported that the mean retention force obtained for PEEK was 4.14 N in lithium disilicate crowns. At the end of 1440 insertion/removal test cycles, there was a significant decrease in the retention values of both groups of the study, which may be due to the clasp deformity in both groups at these cycling intervals. In the same manner, the results of the current study propose that BioHPP RPDs frameworks clasps may lose their retention force value due to multiple deflections, which leads to continuous loss of elasticity [ 14 ]. 

It was found that each group exhibited significant decrease in retention between the initial (0 cycle), 120 cycle, 720 cycles and final retention (1440 cycles) due to significant wear occurred in all groups after simulating 3, 6 and 12 months of RPDs use. This might be a limitation of the present study since only the vertical forces exerted on the RPDs were evaluated and lateral forces affecting the retainer and the horizontal forces, which are to be expected during the chewing process, were not simulated [ 34 ]. As the wear was only simulated in the axial direction, this can be a result of selective wear of certain attachment surfaces. 

This study had several limitations. Primarily, in the *in vitro* study, the movements of the dentures on the stone model are different from clinical situation due to the absence of the supporting soft tissues under the dentures. Additionally, no lubricating material was used in the study protocol. Although in vitro examinations show little correlation with the clinical situations, they might provide comparative evaluation of different materials under standardized conditions.

## Conclusion

Based on the study results, it could be concluded that:

• From the retention point of view, PEEK RPD frameworks fabricated by injection molding technique considered a promising method over CAD/CAM technique milled method.
• BioHPP RPDs frameworks clasps provide an optimal retention value when suitable retentive undercuts are provided.

## Acknowledgement

The authors would like to thank Mr. Khaled shabaan and, Mrs. Ragaa Attayeb for their role in the collection and organization of the article data.

## Conflict of Interest

There are no conflicts of interest.
